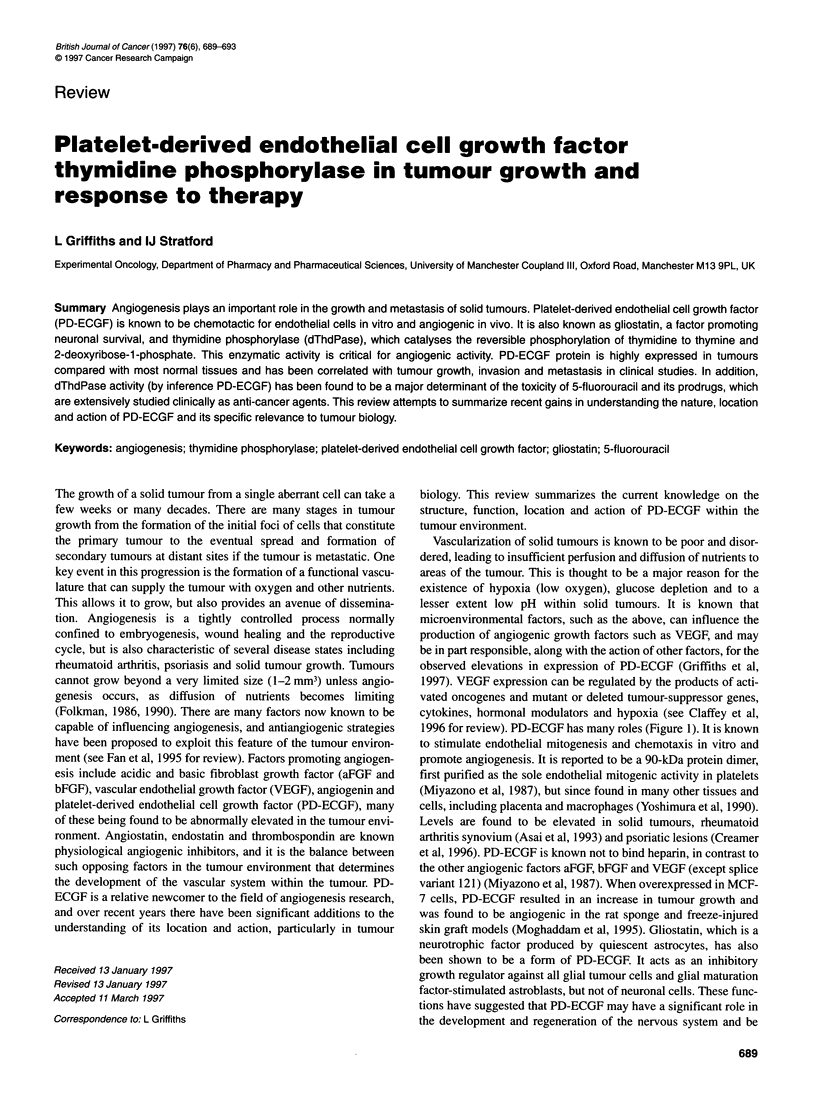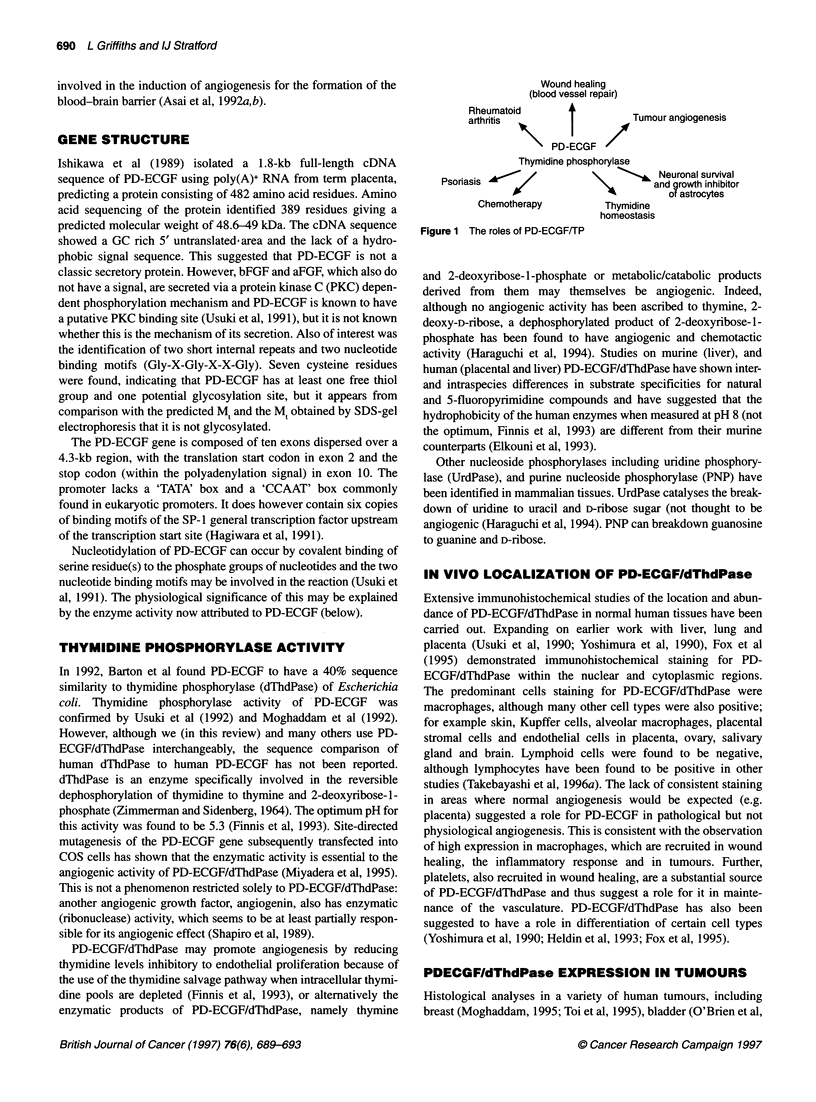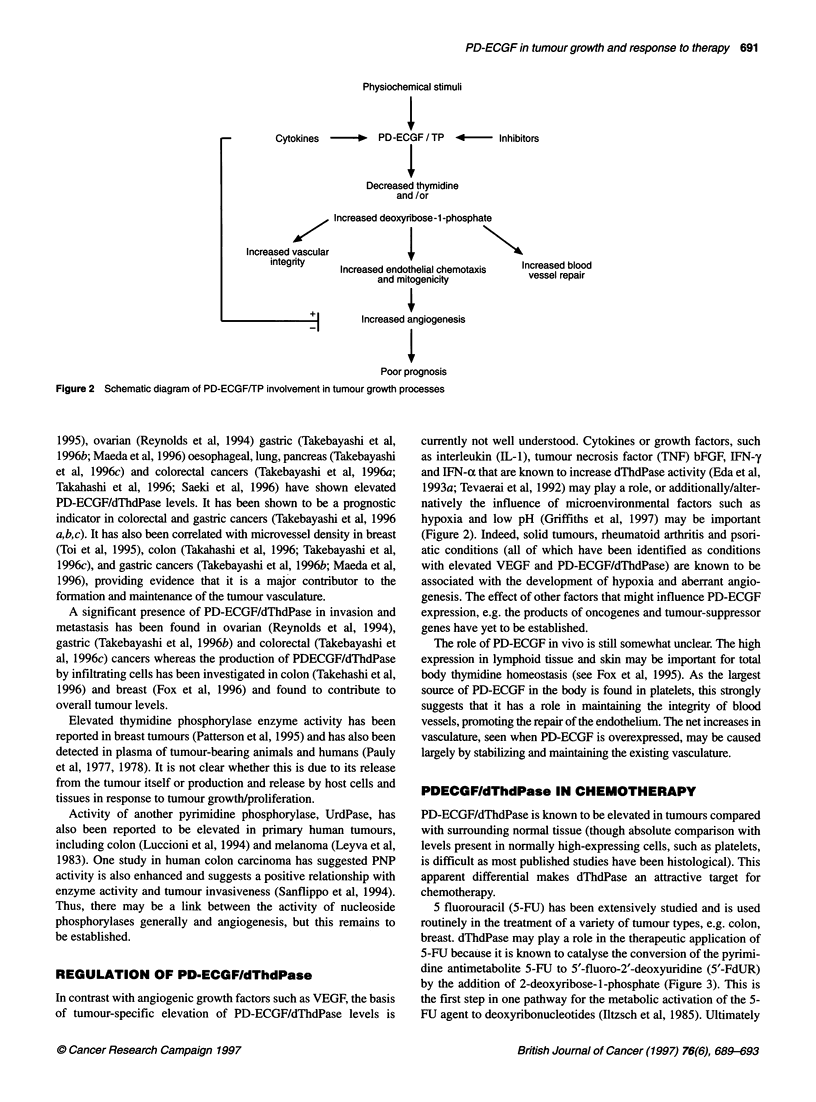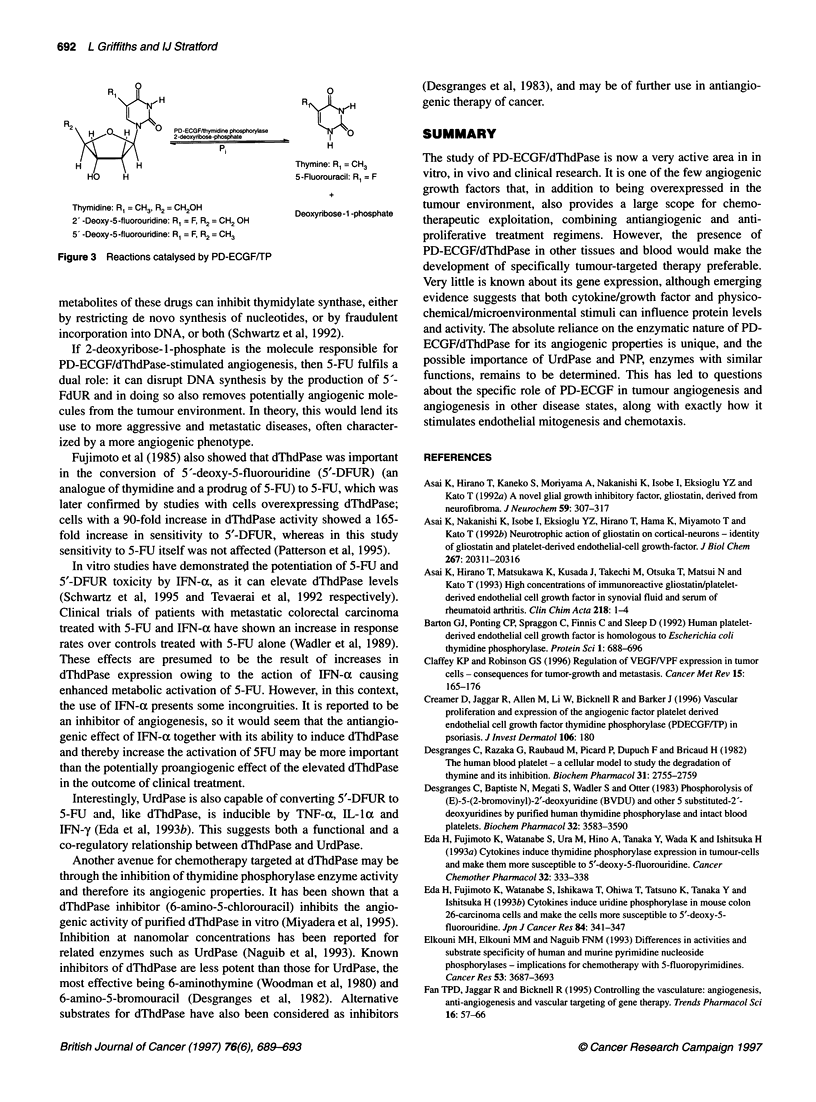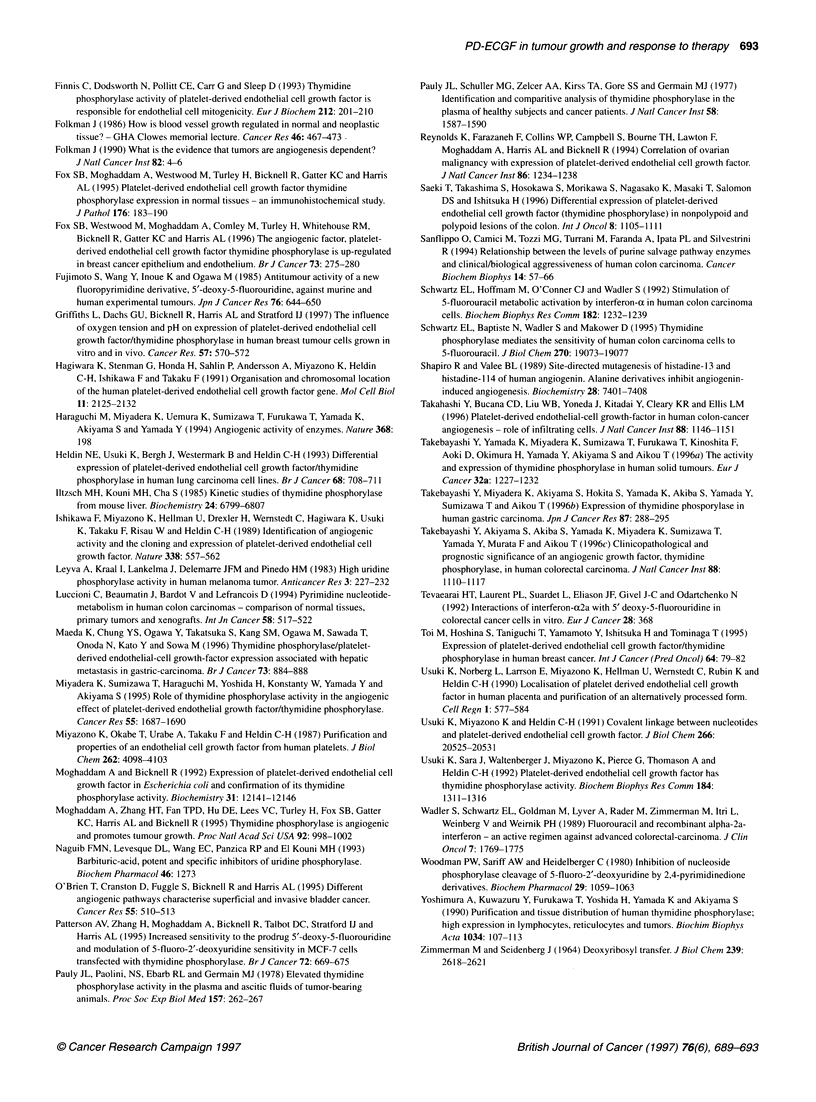# Platelet-derived endothelial cell growth factor thymidine phosphorylase in tumour growth and response to therapy.

**DOI:** 10.1038/bjc.1997.447

**Published:** 1997

**Authors:** L. Griffiths, I. J. Stratford

**Affiliations:** Department of Pharmacy and Pharmaceutical Sciences, University of Manchester Coupland III, UK.

## Abstract

Angiogenesis plays an important role in the growth and metastasis of solid tumours. Platelet-derived endothelial cell growth factor (PD-ECGF) is known to be chemotactic for endothelial cells in vitro and angiogenic in vivo. It is also known as gliostatin, a factor promoting neuronal survival, and thymidine phosphorylase (dThdPase), which catalyses the reversible phosphorylation of thymidine to thymine and 2-deoxyribose-1-phosphate. This enzymatic activity is critical for angiogenic activity. PD-ECGF protein is highly expressed in tumours compared with most normal tissues and has been correlated with tumour growth, invasion and metastasis in clinical studies. In addition, dThdPase activity (by inference PD-ECGF) has been found to be a major determinant of the toxicity of 5-fluorouracil and its prodrugs, which are extensively studied clinically as anti-cancer agents. This review attempts to summarize recent gains in understanding the nature, location and action of PD-ECGF and its specific relevance to tumour biology.


British Journal of Cancer (1997) 76(6), 689–693
© 1997 Cancer Research Campaign

Review

### Platelet-derived endothelial cell growth factor thymidine phosphorylase in tumour growth and response to therapy

**L Griffiths and IJ Stratford**

Experimental Oncology, Department of Pharmacy and Pharmaceutical Sciences, University of Manchester Coupland III, Oxford Road, Manchester M13 9PL, UK

**Summary** Angiogenesis plays an important role in the growth and metastasis of solid tumours. Platelet-derived endothelial cell growth factor (PD-ECGF) is known to be chemotactic for endothelial cells in vitro and angiogenic in vivo. It is also known as gliostatin, a factor promoting neuronal survival, and thymidine phosphorylase (dThdPase), which catalyses the reversible phosphorylation of thymidine to thymine and 2-deoxyribose-1-phosphate. This enzymatic activity is critical for angiogenic activity. PD-ECGF protein is highly expressed in tumours compared with most normal tissues and has been correlated with tumour growth, invasion and metastasis in clinical studies. In addition, dThdPase activity (by inference PD-ECGF) has been found to be a major determinant of the toxicity of 5-fluorouracil and its prodrugs, which are extensively studied clinically as anti-cancer agents. This review attempts to summarize recent gains in understanding the nature, location and action of PD-ECGF and its specific relevance to tumour biology.

**Keywords:** angiogenesis; thymidine phosphorylase; platelet-derived endothelial cell growth factor; gliostatin; 5-fluorouracil

The growth of a solid tumour from a single aberrant cell can take a few weeks or many decades. There are many stages in tumour growth from the formation of the initial foci of cells that constitute the primary tumour to the eventual spread and formation of secondary tumours at distant sites if the tumour is metastatic. One key event in this progression is the formation of a functional vasculature that can supply the tumour with oxygen and other nutrients. This allows it to grow, but also provides an avenue of dissemination. Angiogenesis is a tightly controlled process normally confined to embryogenesis, wound healing and the reproductive cycle, but is also characteristic of several disease states including rheumatoid arthritis, psoriasis and solid tumour growth. Tumours cannot grow beyond a very limited size (1–2 $mm^3$) unless angiogenesis occurs, as diffusion of nutrients becomes limiting (Folkman, 1986, 1990). There are many factors now known to be capable of influencing angiogenesis, and antiangiogenic strategies have been proposed to exploit this feature of the tumour environment (see Fan et al, 1995 for review). Factors promoting angiogenesis include acidic and basic fibroblast growth factor (aFGF and bFGF), vascular endothelial growth factor (VEGF), angiogenin and platelet-derived endothelial cell growth factor (PD-ECGF), many of these being found to be abnormally elevated in the tumour environment. Angiostatin, endostatin and thrombospondin are known physiological angiogenic inhibitors, and it is the balance between such opposing factors in the tumour environment that determines the development of the vascular system within the tumour. PD-ECGF is a relative newcomer to the field of angiogenesis research, and over recent years there have been significant additions to the understanding of its location and action, particularly in tumour biology. This review summarizes the current knowledge on the structure, function, location and action of PD-ECGF within the tumour environment.

Vascularization of solid tumours is known to be poor and disordered, leading to insufficient perfusion and diffusion of nutrients to areas of the tumour. This is thought to be a major reason for the existence of hypoxia (low oxygen), glucose depletion and to a lesser extent low pH within solid tumours. It is known that microenvironmental factors, such as the above, can influence the production of angiogenic growth factors such as VEGF, and may be in part responsible, along with the action of other factors, for the observed elevations in expression of PD-ECGF (Griffiths et al, 1997). VEGF expression can be regulated by the products of activated oncogenes and mutant or deleted tumour-suppressor genes, cytokines, hormonal modulators and hypoxia (see Claffey et al, 1996 for review). PD-ECGF has many roles (Figure 1). It is known to stimulate endothelial mitogenesis and chemotaxis in vitro and promote angiogenesis. It is reported to be a 90-kDa protein dimer, first purified as the sole endothelial mitogenic activity in platelets (Miyazono et al, 1987), but since found in many other tissues and cells, including placenta and macrophages (Yoshimura et al, 1990). Levels are found to be elevated in solid tumours, rheumatoid arthritis synovium (Asai et al, 1993) and psoriatic lesions (Creamer et al, 1996). PD-ECGF is known not to bind heparin, in contrast to the other angiogenic factors aFGF, bFGF and VEGF (except splice variant 121) (Miyazono et al, 1987). When overexpressed in MCF-7 cells, PD-ECGF resulted in an increase in tumour growth and was found to be angiogenic in the rat sponge and freeze-injured skin graft models (Moghaddam et al, 1995). Gliostatin, which is a neurotrophic factor produced by quiescent astrocytes, has also been shown to be a form of PD-ECGF. It acts as an inhibitory growth regulator against all glial tumour cells and glial maturation factor-stimulated astroblasts, but not of neuronal cells. These functions have suggested that PD-ECGF may have a significant role in the development and regeneration of the nervous system and be

*Received 13 January 1997*
*Revised 13 January 1997*
*Accepted 11 March 1997*

*Correspondence to:* L Griffiths involved in the induction of angiogenesis for the formation of the blood–brain barrier (Asai et al, 1992*a*,*b*).

### GENE STRUCTURE

Ishikawa et al (1989) isolated a 1.8-kb full-length cDNA sequence of PD-ECGF using poly(A)$^+$ RNA from term placenta, predicting a protein consisting of 482 amino acid residues. Amino acid sequencing of the protein identified 389 residues giving a predicted molecular weight of 48.6–49 kDa. The cDNA sequence showed a GC rich 5′ untranslated area and the lack of a hydrophobic signal sequence. This suggested that PD-ECGF is not a classic secretory protein. However, bFGF and aFGF, which also do not have a signal, are secreted via a protein kinase C (PKC) dependent phosphorylation mechanism and PD-ECGF is known to have a putative PKC binding site (Usuki et al, 1991), but it is not known whether this is the mechanism of its secretion. Also of interest was the identification of two short internal repeats and two nucleotide binding motifs (Gly-X-Gly-X-X-Gly). Seven cysteine residues were found, indicating that PD-ECGF has at least one free thiol group and one potential glycosylation site, but it appears from comparison with the predicted $M_t$ and the $M_t$ obtained by SDS-gel electrophoresis that it is not glycosylated.

The PD-ECGF gene is composed of ten exons dispersed over a 4.3-kb region, with the translation start codon in exon 2 and the stop codon (within the polyadenylation signal) in exon 10. The promoter lacks a 'TATA' box and a 'CCAAT' box commonly found in eukaryotic promoters. It does however contain six copies of binding motifs of the SP-1 general transcription factor upstream of the transcription start site (Hagiwara et al, 1991).

Nucleotidylation of PD-ECGF can occur by covalent binding of serine residue(s) to the phosphate groups of nucleotides and the two nucleotide binding motifs may be involved in the reaction (Usuki et al, 1991). The physiological significance of this may be explained by the enzyme activity now attributed to PD-ECGF (below).

### THYMIDINE PHOSPHORYLASE ACTIVITY

In 1992, Barton et al found PD-ECGF to have a 40% sequence similarity to thymidine phosphorylase (dThdPase) of *Escherichia coli*. Thymidine phosphorylase activity of PD-ECGF was confirmed by Usuki et al (1992) and Moghaddam et al (1992). However, although we (in this review) and many others use PD-ECGF/dThdPase interchangeably, the sequence comparison of human dThdPase to human PD-ECGF has not been reported. dThdPase is an enzyme specifically involved in the reversible dephosphorylation of thymidine to thymine and 2-deoxyribose-1-phosphate (Zimmerman and Sidenberg, 1964). The optimum pH for this activity was found to be 5.3 (Finnis et al, 1993). Site-directed mutagenesis of the PD-ECGF gene subsequently transfected into COS cells has shown that the enzymatic activity is essential to the angiogenic activity of PD-ECGF/dThdPase (Miyadera et al, 1995). This is not a phenomenon restricted solely to PD-ECGF/dThdPase: another angiogenic growth factor, angiogenin, also has enzymatic (ribonuclease) activity, which seems to be at least partially responsible for its angiogenic effect (Shapiro et al, 1989).

PD-ECGF/dThdPase may promote angiogenesis by reducing thymidine levels inhibitory to endothelial proliferation because of the use of the thymidine salvage pathway when intracellular thymidine pools are depleted (Finnis et al, 1993), or alternatively the enzymatic products of PD-ECGF/dThdPase, namely thymine

**Figure 1** The roles of PD-ECGF/TP

and 2-deoxyribose-1-phosphate or metabolic/catabolic products derived from them may themselves be angiogenic. Indeed, although no angiogenic activity has been ascribed to thymine, 2-deoxy-D-ribose, a dephosphorylated product of 2-deoxyribose-1-phosphate has been found to have angiogenic and chemotactic activity (Haraguchi et al, 1994). Studies on murine (liver), and human (placental and liver) PD-ECGF/dThdPase have shown inter- and intraspecies differences in substrate specificities for natural and 5-fluoropyrimidine compounds and have suggested that the hydrophobicity of the human enzymes when measured at pH 8 (not the optimum, Finnis et al, 1993) are different from their murine counterparts (Elkouni et al, 1993).

Other nucleoside phosphorylases including uridine phosphorylase (UrdPase), and purine nucleoside phosphorylase (PNP) have been identified in mammalian tissues. UrdPase catalyses the breakdown of uridine to uracil and D-ribose sugar (not thought to be angiogenic (Haraguchi et al, 1994). PNP can breakdown guanosine to guanine and D-ribose.

### IN VIVO LOCALIZATION OF PD-ECGF/dThdPase

Extensive immunohistochemical studies of the location and abundance of PD-ECGF/dThdPase in normal human tissues have been carried out. Expanding on earlier work with liver, lung and placenta (Usuki et al, 1990; Yoshimura et al, 1990), Fox et al (1995) demonstrated immunohistochemical staining for PD-ECGF/dThdPase within the nuclear and cytoplasmic regions. The predominant cells staining for PD-ECGF/dThdPase were macrophages, although many other cell types were also positive; for example skin, Kupffer cells, alveolar macrophages, placental stromal cells and endothelial cells in placenta, ovary, salivary gland and brain. Lymphoid cells were found to be negative, although lymphocytes have been found to be positive in other studies (Takebayashi et al, 1996*a*). The lack of consistent staining in areas where normal angiogenesis would be expected (e.g. placenta) suggested a role for PD-ECGF in pathological but not physiological angiogenesis. This is consistent with the observation of high expression in macrophages, which are recruited in wound healing, the inflammatory response and in tumours. Further, platelets, also recruited in wound healing, are a substantial source of PD-ECGF/dThdPase and thus suggest a role for it in maintenance of the vasculature. PD-ECGF/dThdPase has also been suggested to have a role in differentiation of certain cell types (Yoshimura et al, 1990; Heldin et al, 1993; Fox et al, 1995).

### PDECGF/dThdPase EXPRESSION IN TUMOURS

Histological analyses in a variety of human tumours, including breast (Moghaddam, 1995; Toi et al, 1995), bladder (O'Brien et al,

 © Cancer Research Campaign 1997

**Figure 2** Schematic diagram of PD-ECGF/TP involvement in tumour growth processes

1995), ovarian (Reynolds et al, 1994) gastric (Takebayashi et al, 1996*b*; Maeda et al, 1996) oesophageal, lung, pancreas (Takebayashi et al, 1996*c*) and colorectal cancers (Takebayashi et al, 1996*a*; Takahashi et al, 1996; Saeki et al, 1996) have shown elevated PD-ECGF/dThdPase levels. It has been shown to be a prognostic indicator in colorectal and gastric cancers (Takebayashi et al, 1996 *a,b,c*). It has also been correlated with microvessel density in breast (Toi et al, 1995), colon (Takahashi et al, 1996; Takebayashi et al, 1996*c*), and gastric cancers (Takebayashi et al, 1996*b*; Maeda et al, 1996), providing evidence that it is a major contributor to the formation and maintenance of the tumour vasculature.

A significant presence of PD-ECGF/dThdPase in invasion and metastasis has been found in ovarian (Reynolds et al, 1994), gastric (Takebayashi et al, 1996*b*) and colorectal (Takebayashi et al, 1996*c*) cancers whereas the production of PDECGF/dThdPase by infiltrating cells has been investigated in colon (Takehashi et al, 1996) and breast (Fox et al, 1996) and found to contribute to overall tumour levels.

Elevated thymidine phosphorylase enzyme activity has been reported in breast tumours (Patterson et al, 1995) and has also been detected in plasma of tumour-bearing animals and humans (Pauly et al, 1977, 1978). It is not clear whether this is due to its release from the tumour itself or production and release by host cells and tissues in response to tumour growth/proliferation.

Activity of another pyrimidine phosphorylase, UrdPase, has also been reported to be elevated in primary human tumours, including colon (Luccioni et al, 1994) and melanoma (Leyva et al, 1983). One study in human colon carcinoma has suggested PNP activity is also enhanced and suggests a positive relationship with enzyme activity and tumour invasiveness (Sanflippo et al, 1994). Thus, there may be a link between the activity of nucleoside phosphorylases generally and angiogenesis, but this remains to be established.

### REGULATION OF PD-ECGF/dThdPase

In contrast with angiogenic growth factors such as VEGF, the basis of tumour-specific elevation of PD-ECGF/dThdPase levels is currently not well understood. Cytokines or growth factors, such as interleukin (IL-1), tumour necrosis factor (TNF) bFGF, IFN-γ and IFN-α that are known to increase dThdPase activity (Eda et al, 1993*a*; Tevaerai et al, 1992) may play a role, or additionally/alternatively the influence of microenvironmental factors such as hypoxia and low pH (Griffiths et al, 1997) may be important (Figure 2). Indeed, solid tumours, rheumatoid arthritis and psoriatic conditions (all of which have been identified as conditions with elevated VEGF and PD-ECGF/dThdPase) are known to be associated with the development of hypoxia and aberrant angiogenesis. The effect of other factors that might influence PD-ECGF expression, e.g. the products of oncogenes and tumour-suppressor genes have yet to be established.

The role of PD-ECGF in vivo is still somewhat unclear. The high expression in lymphoid tissue and skin may be important for total body thymidine homeostasis (see Fox et al, 1995). As the largest source of PD-ECGF in the body is found in platelets, this strongly suggests that it has a role in maintaining the integrity of blood vessels, promoting the repair of the endothelium. The net increases in vasculature, seen when PD-ECGF is overexpressed, may be caused largely by stabilizing and maintaining the existing vasculature.

### PDECGF/dThdPase IN CHEMOTHERAPY

PD-ECGF/dThdPase is known to be elevated in tumours compared with surrounding normal tissue (though absolute comparison with levels present in normally high-expressing cells, such as platelets, is difficult as most published studies have been histological). This apparent differential makes dThdPase an attractive target for chemotherapy.

5 fluorouracil (5-FU) has been extensively studied and is used routinely in the treatment of a variety of tumour types, e.g. colon, breast. dThdPase may play a role in the therapeutic application of 5-FU because it is known to catalyse the conversion of the pyrimidine antimetabolite 5-FU to 5′-fluoro-2′-deoxyuridine (5′-FdUR) by the addition of 2-deoxyribose-1-phosphate (Figure 3). This is the first step in one pathway for the metabolic activation of the 5-FU agent to deoxyribonucleotides (Iltzsch et al, 1985). Ultimately

© Cancer Research Campaign 1997 

**Figure 3** Reactions catalysed by PD-ECGF/TP

metabolites of these drugs can inhibit thymidylate synthase, either by restricting de novo synthesis of nucleotides, or by fraudulent incorporation into DNA, or both (Schwartz et al, 1992).

If 2-deoxyribose-1-phosphate is the molecule responsible for PD-ECGF/dThdPase-stimulated angiogenesis, then 5-FU fulfils a dual role: it can disrupt DNA synthesis by the production of 5′-FdUR and in doing so also removes potentially angiogenic molecules from the tumour environment. In theory, this would lend its use to more aggressive and metastatic diseases, often characterized by a more angiogenic phenotype.

Fujimoto et al (1985) also showed that dThdPase was important in the conversion of 5′-deoxy-5-fluorouridine (5′-DFUR) (an analogue of thymidine and a prodrug of 5-FU) to 5-FU, which was later confirmed by studies with cells overexpressing dThdPase; cells with a 90-fold increase in dThdPase activity showed a 165-fold increase in sensitivity to 5′-DFUR, whereas in this study sensitivity to 5-FU itself was not affected (Patterson et al, 1995).

In vitro studies have demonstrated the potentiation of 5-FU and 5′-DFUR toxicity by IFN-α, as it can elevate dThdPase levels (Schwartz et al, 1995 and Tevaerai et al, 1992 respectively). Clinical trials of patients with metastatic colorectal carcinoma treated with 5-FU and IFN-α have shown an increase in response rates over controls treated with 5-FU alone (Wadler et al, 1989). These effects are presumed to be the result of increases in dThdPase expression owing to the action of IFN-α causing enhanced metabolic activation of 5-FU. However, in this context, the use of IFN-α presents some incongruities. It is reported to be an inhibitor of angiogenesis, so it would seem that the antiangiogenic effect of IFN-α together with its ability to induce dThdPase and thereby increase the activation of 5FU may be more important than the potentially proangiogenic effect of the elevated dThdPase in the outcome of clinical treatment.

Interestingly, UrdPase is also capable of converting 5′-DFUR to 5-FU and, like dThdPase, is inducible by TNF-α, IL-1α and IFN-γ (Eda et al, 1993*b*). This suggests both a functional and a co-regulatory relationship between dThdPase and UrdPase.

Another avenue for chemotherapy targeted at dThdPase may be through the inhibition of thymidine phosphorylase enzyme activity and therefore its angiogenic properties. It has been shown that a dThdPase inhibitor (6-amino-5-chlorouracil) inhibits the angiogenic activity of purified dThdPase in vitro (Miyadera et al, 1995). Inhibition at nanomolar concentrations has been reported for related enzymes such as UrdPase (Naguib et al, 1993). Known inhibitors of dThdPase are less potent than those for UrdPase, the most effective being 6-aminothymine (Woodman et al, 1980) and 6-amino-5-bromouracil (Desgranges et al, 1982). Alternative substrates for dThdPase have also been considered as inhibitors (Desgranges et al, 1983), and may be of further use in antiangiogenic therapy of cancer.

### SUMMARY

The study of PD-ECGF/dThdPase is now a very active area in in vitro, in vivo and clinical research. It is one of the few angiogenic growth factors that, in addition to being overexpressed in the tumour environment, also provides a large scope for chemotherapeutic exploitation, combining antiangiogenic and antiproliferative treatment regimens. However, the presence of PD-ECGF/dThdPase in other tissues and blood would make the development of specifically tumour-targeted therapy preferable. Very little is known about its gene expression, although emerging evidence suggests that both cytokine/growth factor and physicochemical/microenvironmental stimuli can influence protein levels and activity. The absolute reliance on the enzymatic nature of PD-ECGF/dThdPase for its angiogenic properties is unique, and the possible importance of UrdPase and PNP, enzymes with similar functions, remains to be determined. This has led to questions about the specific role of PD-ECGF in tumour angiogenesis and angiogenesis in other disease states, along with exactly how it stimulates endothelial mitogenesis and chemotaxis.

### REFERENCES

Asai K, Hirano T, Kaneko S, Moriyama A, Nakanishi K, Isobe I, Eksioglu YZ and Kato T (1992*a*) A novel glial growth inhibitory factor, gliostatin, derived from neurofibroma. *J Neurochem* **59**: 307–317

Asai K, Nakanishi K, Isobe I, Eksioglu YZ, Hirano T, Hama K, Miyamoto T and Kato T (1992*b*) Neurotrophic action of gliostatin on cortical-neurons – identity of gliostatin and platelet-derived endothelial-cell growth-factor. *J Biol Chem* **267**: 20311–20316

Asai K, Hirano T, Matsukawa K, Kusada J, Takechi M, Otsuka T, Matsui N and Kato T (1993) High concentrations of immunoreactive gliostatin/platelet-derived endothelial cell growth factor in synovial fluid and serum of rheumatoid arthritis. *Clin Chim Acta* **218**: 1–4

Barton GJ, Ponting CP, Spraggon C, Finnis C and Sleep D (1992) Human platelet-derived endothelial cell growth factor is homologous to *Escherichia coli* thymidine phosphorylase. *Protein Sci* **1**: 688–696

Claffey KP and Robinson GS (1996) Regulation of VEGF/VPF expression in tumor cells – consequences for tumor-growth and metastasis. *Cancer Met Rev* **15**: 165–176

Creamer D, Jaggar R, Allen M, Li W, Bicknell R and Barker J (1996) Vascular proliferation and expression of the angiogenic factor platelet derived endothelial cell growth factor thymidine phosphorylase (PDECGF/TP) in psoriasis. *J Invest Dermatol* **106**: 180

Desgranges C, Razaka G, Raubaud M, Picard P, Dupuch F and Bricaud H (1982) The human blood platelet – a cellular model to study the degradation of thymine and its inhibition. *Biochem Pharmacol* **31**: 2755–2759

Desgranges C, Baptiste N, Megati S, Wadler S and Otter (1983) Phosphorolysis of (E)-5-(2-bromovinyl)-2′-deoxyuridine (BVDU) and other 5 substituted-2′-deoxyuridines by purified human thymidine phosphorylase and intact blood platelets. *Biochem Pharmacol* **32**: 3583–3590

Eda H, Fujimoto K, Watanabe S, Ura M, Hino A, Tanaka Y, Wada K and Ishitsuka H (1993*a*) Cytokines induce thymidine phosphorylase expression in tumour-cells and make them more susceptible to 5′-deoxy-5-fluorouridine. *Cancer Chemother Pharmacol* **32**: 333–338

Eda H, Fujimoto K, Watanabe S, Ishikawa T, Ohiwa T, Tatsuno K, Tanaka Y and Ishitsuka H (1993*b*) Cytokines induce uridine phosphorylase in mouse colon 26-carcinoma cells and make the cells more susceptible to 5′-deoxy-5-fluorouridine. *Jpn J Cancer Res* **84**: 341–347

Elkouni MH, Elkouni MM and Naguib FNM (1993) Differences in activities and substrate specificity of human and murine pyrimidine nucleoside phosphorylases – implications for chemotherapy with 5-fluoropyrimidines. *Cancer Res* **53**: 3687–3693

Fan TPD, Jaggar R and Bicknell R (1995) Controlling the vasculature: angiogenesis, anti-angiogenesis and vascular targeting of gene therapy. *Trends Pharmacol Sci* **16**: 57–66

 © Cancer Research Campaign 1997

Finnis C, Dodsworth N, Pollitt CE, Carr G and Sleep D (1993) Thymidine phosphorylase activity of platelet-derived endothelial cell growth factor is responsible for endothelial cell mitogenicity. *Eur J Biochem* **212**: 201–210

Folkman J (1986) How is blood vessel growth regulated in normal and neoplastic tissue? – GHA Clowes memorial lecture. *Cancer Res* **46**: 467–473

Folkman J (1990) What is the evidence that tumors are angiogenesis dependent? *J Natl Cancer Inst* **82**: 4–6

Fox SB, Moghaddam A, Westwood M, Turley H, Bicknell R, Gatter KC and Harris AL (1995) Platelet-derived endothelial cell growth factor thymidine phosphorylase expression in normal tissues – an immunohistochemical study. *J Pathol* **176**: 183–190

Fox SB, Westwood M, Moghaddam A, Comley M, Turley H, Whitehouse RM, Bicknell R, Gatter KC and Harris AL (1996) The angiogenic factor, platelet-derived endothelial cell growth factor thymidine phosphorylase is up-regulated in breast cancer epithelium and endothelium. *Br J Cancer* **73**: 275–280

Fujimoto S, Wang Y, Inoue K and Ogawa M (1985) Antitumour activity of a new fluoropyrimidine derivative, 5′-deoxy-5-fluorouridine, against murine and human experimental tumours. *Jpn J Cancer Res* **76**: 644–650

Griffiths L, Dachs GU, Bicknell R, Harris AL and Stratford IJ (1997) The influence of oxygen tension and pH on expression of platelet-derived endothelial cell growth factor/thymidine phosphorylase in human breast tumour cells grown in vitro and in vivo. *Cancer Res.* **57**: 570–572

Hagiwara K, Stenman G, Honda H, Sahlin P, Andersson A, Miyazono K, Heldin C-H, Ishikawa F and Takaku F (1991) Organisation and chromosomal location of the human platelet-derived endothelial cell growth factor gene. *Mol Cell Biol* **11**: 2125–2132

Haraguchi M, Miyadera K, Uemura K, Sumizawa T, Furukawa T, Yamada K, Akiyama S and Yamada Y (1994) Angiogenic activity of enzymes. *Nature* **368**: 198

Heldin NE, Usuki K, Bergh J, Westermark B and Heldin C-H (1993) Differential expression of platelet-derived endothelial cell growth factor/thymidine phosphorylase in human lung carcinoma cell lines. *Br J Cancer* **68**: 708–711

Iltzsch MH, Kouni MH, Cha S (1985) Kinetic studies of thymidine phosphorylase from mouse liver. *Biochemistry* **24**: 6799–6807

Ishikawa F, Miyazono K, Hellman U, Drexler H, Wernstedt C, Hagiwara K, Usuki K, Takaku F, Risau W and Heldin C-H (1989) Identification of angiogenic activity and the cloning and expression of platelet-derived endothelial cell growth factor. *Nature* **338**: 557–562

Leyva A, Kraal I, Lankelma J, Delemarre JFM and Pinedo HM (1983) High uridine phosphorylase activity in human melanoma tumor. *Anticancer Res* **3**: 227–232

Luccioni C, Beaumatin J, Bardot V and Lefrancois D (1994) Pyrimidine nucleotide-metabolism in human colon carcinomas – comparison of normal tissues, primary tumors and xenografts. *Int Jn Cancer* **58**: 517–522

Maeda K, Chung YS, Ogawa Y, Takatsuka S, Kang SM, Ogawa M, Sawada T, Onoda N, Kato Y and Sowa M (1996) Thymidine phosphorylase/platelet-derived endothelial-cell growth-factor expression associated with hepatic metastasis in gastric-carcinoma. *Br J Cancer* **73**: 884–888

Miyadera K, Sumizawa T, Haraguchi M, Yoshida H, Konstanty W, Yamada Y and Akiyama S (1995) Role of thymidine phosphorylase activity in the angiogenic effect of platelet-derived endothelial growth factor/thymidine phosphorylase. *Cancer Res* **55**: 1687–1690

Miyazono K, Okabe T, Urabe A, Takaku F and Heldin C-H (1987) Purification and properties of an endothelial cell growth factor from human platelets. *J Biol Chem* **262**: 4098–4103

Moghaddam A and Bicknell R (1992) Expression of platelet-derived endothelial cell growth factor in *Escherichia coli* and confirmation of its thymidine phosphorylase activity. *Biochemistry* **31**: 12141–12146

Moghaddam A, Zhang HT, Fan TPD, Hu DE, Lees VC, Turley H, Fox SB, Gatter KC, Harris AL and Bicknell R (1995) Thymidine phosphorylase is angiogenic and promotes tumour growth. *Proc Natl Acad Sci USA* **92**: 998–1002

Naguib FMN, Levesque DL, Wang EC, Panzica RP and El Kouni MH (1993) Barbituric-acid, potent and specific inhibitors of uridine phosphorylase. *Biochem Pharmacol* **46**: 1273

O'Brien T, Cranston D, Fuggle S, Bicknell R and Harris AL (1995) Different angiogenic pathways characterise superficial and invasive bladder cancer. *Cancer Res* **55**: 510–513

Patterson AV, Zhang H, Moghaddam A, Bicknell R, Talbot DC, Stratford IJ and Harris AL (1995) Increased sensitivity to the prodrug 5′-deoxy-5-fluorouridine and modulation of 5-fluoro-2′-deoxyuridine sensitivity in MCF-7 cells transfected with thymidine phosphorylase. *Br J Cancer* **72**: 669–675

Pauly JL, Paolini, NS, Ebarb RL and Germain MJ (1978) Elevated thymidine phosphorylase activity in the plasma and ascitic fluids of tumor-bearing animals. *Proc Soc Exp Biol Med* **157**: 262–267

Pauly JL, Schuller MG, Zelcer AA, Kirss TA, Gore SS and Germain MJ (1977) Identification and comparitive analysis of thymidine phosphorylase in the plasma of healthy subjects and cancer patients. *J Natl Cancer Inst* **58**: 1587–1590

Reynolds K, Farazaneh F, Collins WP, Campbell S, Bourne TH, Lawton F, Moghaddam A, Harris AL and Bicknell R (1994) Correlation of ovarian malignancy with expression of platelet-derived endothelial cell growth factor. *J Natl Cancer Inst* **86**: 1234–1238

Saeki T, Takashima S, Hosokawa S, Morikawa S, Nagasako K, Masaki T, Salomon DS and Ishitsuka H (1996) Differential expression of platelet-derived endothelial cell growth factor (thymidine phosphorylase) in nonpolypoid and polypoid lesions of the colon. *Int J Oncol* **8**: 1105–1111

Sanflippo O, Camici M, Tozzi MG, Turrani M, Faranda A, Ipata PL and Silvestrini R (1994) Relationship between the levels of purine salvage pathway enzymes and clinical/biological aggressiveness of human colon carcinoma. *Cancer Biochem Biophys* **14**: 57–66

Schwartz EL, Hoffmam M, O'Conner CJ and Wadler S (1992) Stimulation of 5-fluorouracil metabolic activation by interferon-α in human colon carcinoma cells. *Biochem Biophys Res Comm* **182**: 1232–1239

Schwartz EL, Baptiste N, Wadler S and Makower D (1995) Thymidine phosphorylase mediates the sensitivity of human colon carcinoma cells to 5-fluorouracil. *J Biol Chem* **270**: 19073–19077

Shapiro R and Valee BL (1989) Site-directed mutagenesis of histadine-13 and histadine-114 of human angiogenin. Alanine derivatives inhibit angiogenin-induced angiogenesis. *Biochemistry* **28**: 7401–7408

Takahashi Y, Bucana CD, Liu WB, Yoneda J, Kitadai Y, Cleary KR and Ellis LM (1996) Platelet-derived endothelial-cell growth-factor in human colon-cancer angiogenesis – role of infiltrating cells. *J Natl Cancer Inst* **88**: 1146–1151

Takebayashi Y, Yamada K, Miyadera K, Sumizawa T, Furukawa T, Kinoshita F, Aoki D, Okimura H, Yamada Y, Akiyama S and Aikou T (1996*a*) The activity and expression of thymidine phosphorylase in human solid tumours. *Eur J Cancer* **32a**: 1227–1232

Takebayashi Y, Miyadera K, Akiyama S, Hokita S, Yamada K, Akiba S, Yamada Y, Sumizawa T and Aikou T (1996*b*) Expression of thymidine phosporylase in human gastric carcinoma. *Jpn J Cancer Res* **87**: 288–295

Takebayashi Y, Akiyama S, Akiba S, Yamada K, Miyadera K, Sumizawa T, Yamada Y, Murata F and Aikou T (1996*c*) Clinicopathological and prognostic significance of an angiogenic growth factor, thymidine phosphorylase, in human colorectal carcinoma. *J Natl Cancer Inst* **88**: 1110–1117

Tevaearai HT, Laurent PL, Suardet L, Eliason JF, Givel J-C and Odartchenko N (1992) Interactions of interferon-α2a with 5′ deoxy-5-fluorouridine in colorectal cancer cells in vitro. *Eur J Cancer* **28**: 368

Toi M, Hoshina S, Taniguchi T, Yamamoto Y, Ishitsuka H and Tominaga T (1995) Expression of platelet-derived endothelial cell growth factor/thymidine phosphorylase in human breast cancer. *Int J Cancer (Pred Oncol)* **64**: 79–82

Usuki K, Norberg L, Larrson E, Miyazono K, Hellman U, Wernstedt C, Rubin K and Heldin C-H (1990) Localisation of platelet derived endothelial cell growth factor in human placenta and purification of an alternatively processed form. *Cell Regn* **1**: 577–584

Usuki K, Miyazono K and Heldin C-H (1991) Covalent linkage between nucleotides and platelet-derived endothelial cell growth factor. *J Biol Chem* **266**: 20525–20531

Usuki K, Sara J, Waltenberger J, Miyazono K, Pierce G, Thomason A and Heldin C-H (1992) Platelet-derived endothelial cell growth factor has thymidine phosphorylase activity. *Biochem Biophys Res Comm* **184**: 1311–1316

Wadler S, Schwartz EL, Goldman M, Lyver A, Rader M, Zimmerman M, Itri L, Weinberg V and Weirnik PH (1989) Fluorouracil and recombinant alpha-2a-interferon – an active regimen against advanced colorectal-carcinoma. *J Clin Oncol* **7**: 1769–1775

Woodman PW, Sariff AW and Heidelberger C (1980) Inhibition of nucleoside phosphorylase cleavage of 5-fluoro-2′-deoxyuridine by 2,4-pyrimidinedione derivatives. *Biochem Pharmacol* **29**: 1059–1063

Yoshimura A, Kuwazuru Y, Furukawa T, Yoshida H, Yamada K and Akiyama S (1990) Purification and tissue distribution of human thymidine phosphorylase; high expression in lymphocytes, reticulocytes and tumors. *Biochim Biophys Acta* **1034**: 107–113

Zimmerman M and Seidenberg J (1964) Deoxyribosyl transfer. *J Biol Chem* **239**: 2618–2621

© Cancer Research Campaign 1997